# Dynamic Response and Adaptation of Grassland Ecosystems in the Three-River Headwaters Region under Changing Environment: A Review

**DOI:** 10.3390/ijerph20054220

**Published:** 2023-02-27

**Authors:** Yaowen Kou, Quanzhi Yuan, Xiangshou Dong, Shujun Li, Wei Deng, Ping Ren

**Affiliations:** 1 Institute of Geography and Resources Science, Sichuan Normal University, Chengdu 610101, China; 2 Sustainable Development Research Center of Resource and Environment of Western Sichuan, Chengdu 610066, China; 3 Key Lab of Land Resources Evaluation and Monitoring in Southwest, Ministry of Education, Sichuan Normal University, Chengdu 610066, China

**Keywords:** Three-River Headwaters Region, alpine meadow, climate change, grassland degradation, grassland restoration, sustainable grazing

## Abstract

The Three-River Headwaters Region (TRHR) is crucial to the sustainable development of China and Southeast Asia. The sustainability of grassland ecosystems in the region has been seriously challenged in recent years. This paper reviewed the changes in the grasslands of the TRHR and their responses to climate change and human activities. The review showed that accurate monitoring of grassland ecological information is the basis for effective management. Although alpine grassland coverage and the above-ground biomass of the alpine grassland have generally increased in the region over the past 30 years, the degradation has not been fundamentally curbed. Grassland degradation substantially reduced topsoil nutrients and affected their distribution, deteriorated soil moisture conditions, and aggravated soil erosion. Grassland degradation led to loss of productivity and species diversity, and this is already harming the well-being of pastoralists. The “warm and wet” trend of the climate promoted the restoration of alpine grasslands, but widespread overgrazing is considered as one of the main reasons for grassland degradation, and related differences still exist. Since 2000, the grassland restoration policy has achieved fruitful results, but the formulation of the policy still needs to integrate market logic effectively and strengthen the understanding of the relationship between ecological protection and cultural protection. In addition, appropriate human intervention mechanisms are urgently needed due to the uncertainty of future climate change. For mildly and moderately degraded grassland, traditional methods are applicable. However, the severely degraded “black soil beach” needs to be restored by artificial seeding, and the stability of the plant–soil system needs to be emphasized to establish a relatively stable community to prevent secondary degradation.

## 1. Introduction

**Figure 1:**
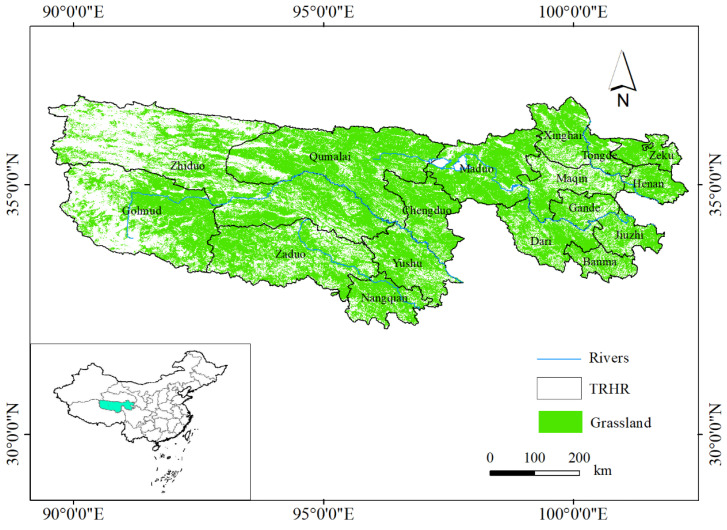
TRHR grassland distribution map [5].

The Three-River Headwaters Region (TRHR) is the source of the Yangtze River, the Yellow River, and the Lancang River. Located in the southern part of Qinghai Province, it is a veritable “Chinese Water Tower” (Figure 1). It is a sensitive area and an important starting area for global climate change. It is one of the most sensitive and fragile ecosystems, and crucial to the sustainable development of Southeast Asian countries [1,2]. It also occupies a special position in Chinese animal husbandry [3]. Grassland is the most dominant type of cover in TRHR and provides the most ecosystem services [4].

TRHR is not only the largest nature reserve in China, but also the area with the highest concentration of biodiversity in high-altitude areas in the world. Its ecosystem is typical and representative of the Qinghai–Tibet Plateau. As the most important germplasm resource bank of plateau organisms in China, its protection value is of great importance to China and the world [6]. The quality and function of TRHR grassland ecosystems directly affect China’s ecological security, biodiversity, water supply, climate system stability, and carbon balance, and play a unique, irreplaceable role in the ecological security of China and the world. Coordinating ecological protection and economic development is crucial. Given that most Tibetans are almost entirely dependent on livestock grazing, grassland ecosystems have enormous livelihood, cultural, and spiritual significance to the local area; ecological and social sustainability need to be achieved in a more balanced manner [7]. Therefore, its ecological environment is the focus of academic research. How to utilize ecological resources rationally, promote the coordinated, sustainable development of regional ecological economy has become the focus of common attention of experts and scholars.

However, single and continuous alpine meadows combined with a harsh natural environment are the reasons for the fragile nature of TRHR alpine grasslands [8]. Under climate change conditions, TRHR agricultural economic development, overgrazing, grassland abandonment, and construction have led to grassland degradation [9,10,11]. Important service functions of grassland ecosystems have been degraded, especially ecological service functions. Production services and herders’ livelihoods have also been affected. The sustainability of TRHR grassland ecosystems is being challenged [12]. The importance of addressing the challenges should be fully recognized, which is crucial for the sustainable development of TRHR as well as the middle and lower reaches.

Given the unique geography and strategic location of TRHR, reviewing the scientific understanding of grassland ecosystems in the region, which is critical for innovative approaches to maintaining ecosystem services and improving the resilience of grassland ecosystems to global change, is an urgent need. For its critical, unique ecosystem, innovative theories are desperately needed. Therefore, this paper reviews the dynamic monitoring of grassland in the TRHR under changing environment, TRHR grassland degradation and its quantitative assessment, ecological effects of grassland degradation, driving factors and mechanisms of grassland degradation, and grassland ecosystem protection policies and restoration measures for degraded grasslands.

## 2. Dynamic Monitoring of the Three-River Headwaters Region Grassland under Changing Environment

### 2.1. Normalized Difference Vegetation Index Approach for Adaptation to Complex Vegetation Dynamics

The normalized difference vegetation index (NDVI), derived from remote sensing, is a powerful indicator of vegetation cover and biomass, and quantifies vegetation by measuring the difference between near-infrared and red light. Owing to its long-term data series, simplicity, and ease of use, the TRHR NDVI has also been widely studied by researchers to assess environmental change and ecosystem responses.

These NDVI data are mainly derived from sensors such as AVHRR [13,14,15,16], MODIS [17,18,19], TM, and ETM [13]. The models of NDVI trend analysis can be divided into linear and nonlinear models. Linear model studies such as Bai, Guo, Degen, Ahmad, Wang, Zhang, Li, Ma, Huang, Zeng, Qi, Long, and Shang [13] used multiple high-resolution satellite data to determine the annual average NDVI, and the results revealed that the NDVI of TRHR showed a weak growth trend from 2000 to 2015. Grassland NDVI changed between 0.43 and 0.50, and the variation range was between 0.23 and 0.27. Climate warming has promoted the growth of TRHR vegetation since the 21st century; however, no evident trend in rainfall is seen, and vegetation changes show partition and segmentation effects. A study by Qian, Fu, and Pan [15] based on data from the US Earth Resources Observation System also supported this conclusion. Grassland NDVI increased after 1994, and the increase has been larger since 2004. However, the study (based on 8 km × 8 km pixel resolution images) by Gillespie, Madson, Cusack, and Xue [16] showed that during 1982–2015, no changes in NDVI were detected in TRHR, but regional differences were considerable, with an increase in the western region and a decrease in the eastern region. Similarly, the trend analysis results of Zheng, Han, Huang, Fassnacht, Xie, Lv, and Chen [14] based on the linear regression model showed no apparent positive and negative trends in the annual NDVI time series from 2001 to 2013.

However, due to the complexity of the ecosystem and the uncertainty of the dynamic driving mechanism of vegetation under climate change, the above-mentioned simple linear research methods may not reveal the complex nonlinear relationship in the dynamic process of vegetation. To explore the nonlinear relationship of the vegetation dynamic process fully, Zheng et al. [14] used stepwise cluster analysis to explore the relationship between NDVI and climate factors. The study showed that precipitation is the main climatic factor leading to different NDVI values in TRHR, and a one-month lag effect exists. However, the increase in temperature in the nongrowing season led to an increase in the annual NDVI value, which may be due to the increase in water demand for vegetation growth. Shen, An, Feng, Ye, Zhu, and Li [17] used Detecting Breakpoints and Estimating Segments in Trend (DBEST) to detect changes in TRHR NDVI while describing long-term trends and detailed short-term fluctuations. The results showed that TRHR vegetation generally recovered during 2000–2015, although the progressive fluctuations of the trend may indicate a potential risk. In addition, Ge, Meng, Liang, Feng, Gao, Yang, Huang, and Xie [18] used MODIS-NDVI data (250 m spatial resolution) and machine learning models such as support vector machine regression (SVMR) models to study the source area of the Yellow River. The results showed that 59.9% of the grasslands increased their vegetation cover during 2001–2016, which also supported the conclusion that the coverage increased.

Most of the studies mentioned above showed an increasing trend in NDVI, especially since the 21st century, but differences were observed in the results. Among the six articles that studied the changing trend of NDVI (four using AVHRR [13,14,15,16] and two using MODIS [17,18]), except for two using AVHRR that showed no substantial change in the 10 years around 2000, the remaining four showed growth. This finding may be due to the different resolutions of the sensors as well as the datasets and the spatiotemporal resolutions. The NDVI of TRHR has evident regional differences [19] and a high degree of spatiotemporal variability. Human-induced fragmentation of the landscape has contributed to this regional disparity [13]. Moreover, grassland growth changes have noticeable seasonal and interannual changes, so an appropriate monitoring time and time scale must be selected to pay attention to the consideration of growth change characteristics. Further projections suggested TRHR vegetation may show an increasing trend until the end of the 21st century [14]. The possibility of mutation in the future still exists, grassland degradation may still occur [17], and continuous dynamic monitoring is still required to prevent the occurrence of degradation risks.

Current research on TRHR grassland dynamic monitoring based on NDVI is still not deep enough. Complex non-linear vegetation dynamics urgently need to be studied to address potential challenges in the context of climate change. Some scholars have combined complex nonlinear models such as machine learning and Google Earth Engine to conduct research [14,18], which will improve the accuracy and efficiency of information extraction. Currently, many NDVI time series datasets have different spatiotemporal resolutions. In the future, making full use of data fusion and reconstruction technology will be necessary to overcome the problem that long-term high-resolution data cannot be obtained due to the accuracy variance between different satellite data [20]. Google Earth Engine, as the leading machine learning and cloud computing platform, can be used to improve the accuracy and efficiency of NDVI products greatly. In addition, changes in NDVI cannot reflect the succession of vegetation types and structures, so a more accurate analysis of ecosystem changes needs to be further explored in combination with various aspects.

### 2.2. Accurate Modeling Covering Data Based on Machine Learning

Grassland cover and alpine grassland aboveground biomass (AG-AGB) are important for the restoration and management of TRHR grassland ecosystems. Vegetation coverage is an important index to describe vegetation changes [21,22]. Remote sensing is the only effective means to estimate grassland coverage and monitor its long-term dynamic changes in a wide range of harsh environments.

Two methods are used in extracting grassland coverage from satellite remote sensing data: spectral mixture analysis and the empirical model. High-resolution satellite imagery over large areas is limited by high cost and weather conditions, whereas medium-resolution satellite images are more suitable for large-area scales due to their wide coverage and higher temporal resolution. However, studies have found that when using MODIS data to extract its endmember vegetation index (VI), the pixel dichotomy model is not suitable for simulating the grassland coverage of TRHR [18]. Ai et al. [23] also showed the overall accuracy coverage of the MA method is lower than that of random forest classification (RFC), regression analysis (RA), and SVMR.

Empirical models are also one of the common methods for determining vegetation coverage. The empirical formula is used to estimate vegetation cover and has been well verified in practice [24]. VI is very well applied in this regard, but due to environmental differences in different regions, the performance of different vegetation indices varies. In simulating TRHR grassland coverage, NDVI performed better, whereas enhanced vegetation index (EVI) was second, possibly because EVI mainly solves the problem of NDVI saturation in high biomass areas, whereas TRHR does not have such saturation [18]. Moreover, using a single VI-based model often yields good results for only the specified area [25]. Multiple regression models further constrain the model by adding more correlated other variables and are better than univariate VI models [18]. Machine learning models are a more advanced approach compared with traditional linear regression and nonlinear regression and has become a current research trend. Many scholars have compared the prediction effects of different models in TRHR. For example, Ge, Meng, Liang, Feng, Gao, Yang, Huang, and Xie [18] took the source area of the Yellow River as the research area and found that the support vector machine (SVM) model is the optimal model after comparing multiple models (pixel binary model, univariate VI model, multivariate model, and SVM model). From 2001 to 2016, the annual maximum grassland coverage of 59.9% of the grassland area in the region showed an increasing trend. However, the average annual maximum grassland coverage rate in 2016 generally showed an increasing trend from west to east and from north to south. The regions with substantially increased grassland coverage were mostly located in low grass cover areas, whereas the areas with reduced grass coverage were mostly located in high grass cover areas. Similar to this result, Ai, An, Chen, and Huang [23] compared four commonly used alpine grassland coverage estimation methods, including RFC, RA, multiendmember spectral mixture analysis, and SVMR, and showed the SVMR-based grassland coverage estimation accuracy was similar to that of the RFC method, and the stability was better. In addition, Liu et al. [26] showed that the overall recovery trend of TRHR grassland coverage increased by 0.91% between 2000 and 2016. Classification and regression trees (CART) and random forest (RF) methods demonstrate higher accuracy and stability than Bayesian, and SVM. When precipitation is higher, the change of grassland cover at the same altitude and temperature is quite different. Under similar altitude and precipitation conditions, grassland coverage increases considerably with temperature rising. However, the coverage of some grasslands still decreases substantially, and the standard deviation of the change is relatively high, which is mainly affected by the aspect, slope, or altitude of these areas.

Most of these studies showed that the overall vegetation cover of TRHR is improving [13]. However, the changes of grassland cover in the TRHR area are complex and unstable. Therefore, the impact of subsequent warm–wet processes on vegetation will be difficult to predict [26] and should be monitored continuously. Most of these monitoring methods only consider changes in overall grassland coverage, while ignoring species-related factors. However, the upward trend in grassland cover can often be the result of severe invasion by weeds. To reflect the real situation of grassland, Ai, An, Chen, and Huang [23] classified grassland plant types into native plants and noxious weeds, and generated a distribution map based on their spectral difference bands, indicating that the distribution of native plant species is generally dominant. Therefore, future research should further consider changes in grassland structure. In addition, these studies still have some common problems, such as the incomplete matching of the spatial difference position between sample plots and satellite data, the uncertainty of climate data, geographical location, topography, and soil data in the research, and the limitations of the model itself. With the increase in variable factors in the future, different models need to be explored further.

### 2.3. Modeling AG-AGB Data Based on Remote Sensing Data

Grassland aboveground biomass (AGB) can characterize grassland attributes and grassland quality. It is one of the important indicators to study grassland ecosystem health, ecological service value, and grassland degradation [27,28,29]. Two main methods are used for monitoring AG-AGB, namely, traditional field measurement and remote sensing image-driven estimation. Traditional terrestrial methods estimate biomass by sampling in the field [25]. Field sampling can obtain accurate AGB, but its cost is relatively high, and the spatial difference is insufficiently considered [30].

Remote sensing-based methods use the relationship between spectrum, environment, and AGB to build a model to evaluate AGB [19]. The selection of indicators and models is very important for estimation. The accuracy of the model estimation depends on the selection of indicators. The increase in the number of indicators inevitably improves the simulation quality of the model, but it reduces the work efficiency and limits the improvement of the model accuracy. However, the screening of key indicators in specific regions is still challenging. In the actual research of TRHR, the selection of indicators in different studies varies greatly [19,29,31,32]. Zhao, Zhou, Peng, Hu, Ma, Xie, Wang, Liu, and Liu [29] focused on the selection of indicators and identified six items (EVI, radiation, altitude, B5/B7, latitude, and precipitation) out of 33 items that are of great relevance for estimating AG-AGB. In terms of model selection, the prediction accuracy of the AG-AGB model constructed by the machine learning algorithm is higher than that of the traditional multiple regression model [25,31]. In the model-based research of TRHR, many scholars compared the models constructed by multiple algorithms. The results showed that the prediction of AG-AGB by the RF model was better than the models constructed by multiple linear regression, backpropagation artificial neural network (BP-ANN), SVM, Cubist, and CART. [29,31,32]. RF models also exhibited higher stability and accuracy [26]. Most studies showed a gradual increase in TRHR AG-AGB from northwest to southeast, and an overall increase from 2000 onward [19,25,29,31]. However, some studies have also shown that the interannual changes of grassland AGB in most areas of TRHR from 2000 to 2018 were not evident [32]. Some studies have also shown that the trend of change varied in different regions, with TRHR far east (Zeku, Henan) and far southwest (parts of Golmud and Yushu) AG-AGB increased considerably (16.5%), whereas Zhiduo northwest AG-AGB decreased substantially (3.8%) [25].

The monitoring results of grassland in the TRHR area mostly showed an increase in grassland coverage and AG-AGB. Many researchers attributed the increasing trend to a combination of climate change and human factors. Most of these monitoring methods only considered changes in overall grassland coverage or AG-AGB and ignored changes in grassland structural composition. However, severe invasion by noxious weeds may lead to an increase in grassland biomass rather than a decrease. Therefore, to have a clearer understanding of the changes in grassland ecosystems, future research needs to pay attention to the related changes in the composition of grass species (Table 1). In addition, the full use of drone technology further improves the model research. Furthermore, studies mostly focus on data-driven empirical models while studies on process-based models are conspicuously missing in TRHR AGB simulation studies. This situation is detrimental to the understanding of ecosystem mechanisms, possibly due to the large variability in process model simulation results [33] and the simplicity and effectiveness of data-driven models.

In terms of TRHR grassland remote sensing, accurate information of grassland ecology is the basis for effective management. Therefore, developing a comprehensive grassland monitoring model in the future, establishing automatic interpretation, developing more efficient image processing algorithms, and combining artificial intelligence technology to achieve large-scale, real-time multi-source heterogeneity are necessary. This development is also a prerequisite for precision livestock farming.

## 3. Degradation of the Three-River Headwaters Region Grassland

Multiple studies have shown that the TRHR grassland ecosystem is degraded to varying degrees, which is proven by field surveys and satellite images. In the middle and late 1970s, the grassland degradation of TRHR began to form. From the 1970s to the 1990s, grassland degradation continued, and different regions showed distinct patterns [34,35]. However, some studies have also shown that after 1990, the increase in grassland coverage and AG-AGB of TRHR was greater than the decrease, showing an overall increasing trend, and the regional macro-ecological environment improved [36,37]. At the watershed scale, in recent years, the grassland in the headwater of the Yellow River has recovered relatively well, followed by the grassland in the headwater of the Yangtze River, and the grassland in the Lancang River has a poor status [38]. From the perspective of the spatial distribution of grassland changes, a general recovery trend is in the southeastern and central regions of TRHR, whereas grassland quality deteriorates in the northwest of TRHR [25,39].

However, the restoration of TRHR grasslands is partial and temporary, and does not reflect the overall or fundamental improvement; grassland degradation has not been fundamentally suppressed [40,41]. Most of the areas with larger increases in grassland coverage originally had lower grassland coverage, and most areas with decreased grassland coverage originally had higher grassland coverage [18]. Moreover, grasslands recover slowly and degrade fast [13]. Some areas of TRHR (especially the high-altitude areas in the northwest) still have noticeable degradation [42,43,44,45], and the degree of desertification and salinization is still expanding [46]. Liu et al. [47] used linear RA and Hurst index analysis to reveal that the vegetation coverage increased in the northern part of TRHR and decreased in the southern part during 2000–2011. The uncertainty of grassland changes reflects the nature of the grassland in this region, which is prone to mutation and fragility. Recent studies have also shown that the area of extremely degraded grassland in this region accounts for 5.68% of the TRHR area [48], which further illustrates the severely unhealthy status of the TRHR grassland.

### 3.1. Quantitative Assessment of Grassland Degradation

The diagnosis of grassland ecosystem degradation degree is the premise of grassland restoration [49]. Quantitative studies on TRHR grassland degradation can be mainly divided into two categories. The first type is mainly about the establishment of grassland degradation classification standards and degradation assessment in small-scale experimental areas. The second category is an overall assessment of grassland degradation in the entire TRHR.

The grading standard formulation and degradation assessment for TRHR grassland was first established by Ma et al. [50] and integrated some visibility indicators such as grassland coverage, plant height, AG-AGB, and proportion of palatable plants to classify grassland degradation into five grades: no degradation, mild, moderate, severe, and extreme. Mildly and moderately degraded grasslands are generally distributed in summer pastures and transitional pastures. Severely and extremely degraded grasslands are mostly distributed near residential sites or at the center of drinking water points. The terrain is generally gentle, and most of them are winter pastures. To quantify the degree of alpine meadow degradation further, Wen et al. [51] constructed a comprehensive evaluation system based on Ma, Lang, Li, Shi and Dong [50] to quantify the degree of grassland degradation based on the visible indicators to define the grassland degradation index (GDI). It is a good solution to the problem that the survey data does not conform to the specification very well. Using GDI to evaluate the TRHR Maqin alpine grassland found the dominant and sub-dominant grassland species have changed greatly at different degradation levels.

However, grassland degradation is a complex ecological process, which includes not only vegetation degradation but also soil changes. In addition to visible indicators, invisible indicators (such as underground biomass, nutrients in soil) are important parameters reflecting the degradation of grassland ecosystems [52]. A comprehensive understanding of the mechanisms of degradation processes is the basis for monitoring and evaluating grassland degradation. Therefore, to assess grassland degradation more scientifically, the vegetation status should be considered, and the grading, definition and assessment of grassland degradation should be carried out from the perspective of the vegetation–soil system. Only in this way can it better serve the restoration practice of degraded grasslands. Plant species diversity and soil nutrients are important predictors of different degradation stages of alpine meadows, and severe degradation leads to the migration of alpine meadow plant communities [53]. Therefore, soil–plant systems must be analyzed from the perspective of a multidisciplinary strategy [54]. Urease, ratio of microbial biomass nitrogen (MBN) to total nitrogen (TN), hydrolase and soil organic carbon (SOC) are the most important indicators for evaluating soil quality. The ratio of microbial biomass carbon (MBC)/MBN is a key factor affecting grasslands above moderately degraded levels. In extremely degraded grasslands, almost all parameters are key factors, which means human disturbance has a substantial impact on soil quality [55]. Li, Dong, Wen, Wang and Wu [55] comprehensively considered the physical and chemical properties of soil and soil organisms, constructed a systematic index to apply it to soil quality evaluation of TRHR plateau alpine grassland under different disturbance intensities, and divided grassland into three categories: non-degraded grassland with high soil quality index (SQI), moderately degraded grassland with medium SQI, and severely degraded grassland with low SQI. Furthermore, Lin, Li, Xu, Zhang, Du, Liu, Guo and Cao [52] divided the entire degradation succession of the Tibetan alpine Kobresia grasslands into six stages. Their study showed that easily observable features such as plant functional group (PFG) type and mattic epipedon state are associated with less observable features such as root state. Therefore, PFG type, root system, and soil status can measure the degradation level of grassland ecosystems. This situation will help determine the grassland degradation level more easily, so the grassland can be protected more reasonably.

Many scholars have also made overall degradation assessments on TRHR grasslands. Owing to the heavy workload and long cycle of field surveys, researchers mainly use remote sensing and related auxiliary data for large-scale degradation monitoring. To assess the degree of overall grassland degradation in TRHR, An, Wang, Feng, Wu, Wang, Wang, Shen, Lu, Quaye-Ballard, Chen, and Zhao [44] comprehensively considered topography, hydrothermal factors, and soil factors, and divided grassland into 20 grassland productivity units. The grassland degradation level was measured by the change in net primary productivity (NPP) of grassland using the grassland productivity unit technique. The grassland degradation degree in this area was 32.86% in 1990, 36.7% in 2004, and increased by 3.84% in 15 years. Banma, Gande, Henan, Jiuzhi, Tongde, and Zeku were the least degraded in the eastern region of TRHR. The Qumalai degradation level was relatively highest. In 1990, the proportion of degraded grassland in Qumalai reached 63.33%, and the proportion increased to 77.47% in the following 14 years. Maduo and Chengduo were relegated by more than 40%. However, because the interannual variation of AG-AGB may be the result of climatic factors or grassland degradation, the influence of climatic factors needs to be corrected in the relevant assessment of grassland status based on temporal and spatial changes of AG-AGB [38]. Moreover, the climate use efficiency index (CUE) index combined with local climatic conditions can show the degradation of soil. Extreme weather events are also an important factor, as they can lead to degradation of grassland ecosystems in a short period [56]. Therefore, CUE and related indices have been widely used in assessing land degradation in arid and semi-arid regions [57]. Some scholars also used CUE to judge the dynamics of TRHR grassland ecosystems. An, Zhang, Sun, Wang, Shen, Wang, Xing, Huang, and Fan [39] proposed a new CUE index to monitor grassland changes by comprehensively considering a series of climatic factors closely related to vegetation growth and their coordinated climatic factors. The results showed that during the 31 years from 1982 to 2012, grassland degradation and restoration coexisted, accounting for 20.49% and 23.89%, respectively. Zhang, Zhang, Wang, An, and Li [38] further combined CUE with vegetation NPP, grassland coverage, and surface bare rate to construct a more complete evaluation index to evaluate regional grassland dynamics. The results showed that from 2001 to 2016, the headwater of the Yellow River had high NPP, grassland vegetation coverage, and CUE, and a low degree of desertification; the headwater of the Yangtze River had low NPP, grassland vegetation coverage, and CUE, and a high degree of desertification. During this period, the vegetation coverage and VI of TRHR grassland showed an upward trend, and the bald spot rate and NPP showed a decreasing trend. Owing the limitation of TRHR’s harsh natural conditions, conducting long-term multi-point positioning observations is very difficult, so uncertainties are still observed in the monitoring results of the overall situation of grassland degradation. Moreover, changes in plant species in grassland ecosystems are seldom considered in assessing overall TRHR degradation. Therefore, hyperspectral remote sensing data can be used to consider this aspect in the future.

### 3.2. Ecological Effects of Grassland Degradation

The degradation of the TRHR alpine grassland ecosystem substantially affects the service functions of the grassland ecosystem, and the impact of grassland degradation can be reflected in the aspects of ecology, production and livelihoods [58].

Ecological functions are inherent in the system and are the bases for the maintenance and development of the system. From an ecological point of view, grassland degradation in TRHR has greatly weakened ecological functions such as carbon sinks, climate regulation, soil conservation, water conservation, biodiversity conservation, and nutrient cycling [53,58]. The TRHR grassland ecosystem has a considerable carbon sink role [59]. Grassland degradation can lead to poor soil quality or even to desert grasslands in the region, resulting in changes in seed banks, and changes in soil properties such as soil moisture, SOC, TN, and soil bulk density, soil microorganisms, and soil enzymes [39,49]. With soil degradation in this area, the soil fertility of the uppermost soil layer within 30 cm decreased considerably [53]. In different degradation succession stages, the correlation between the biomass of the alpine meadow community and soil nutrients (TN, available nitrogen, total phosphorus, available phosphorus, SOC, and soil MBC) in the previous succession stage was positive. With grassland degradation, SOC and TN showed a downward trend, and the distribution of SOC was greatly affected. The proportion of light fraction carbon in total organic carbon (TOC) gradually decreased, Whereas the proportion of heavy fraction carbon in TOC gradually increased [60]. A study conducted at TRHR showed that non-degraded grassland had the highest SOC content. Compared with the non-degraded grassland, the SOC content decreased by 21.89%, 38.30%, and 43.15% with the development of degradation. The TN content in the non-degraded grassland was also higher than that in any of the degraded grasslands (0.908, 0.786, and 0.769 kg·m^−2^ for moderate, severe, and extreme degradation, respectively) [61]. The loss of SOC caused by grassland degradation will have a positive feedback on climate warming, which will intensify the warming.

Vegetation is the main factor responsible for increased soil erosion [62]. The average soil erosion modulus decreased linearly with the increase in vegetation coverage (R^2^ ≥ 0.997). The average erosion modulus of the severely degraded meadow is 2.23 times that of the mildly degraded meadow, and the maximum erosion modulus is 2.96 × 106 kg·km^−2^·a^−1^. About 121.28 × 10^7^ t of soil and water conservation capacity is lost due to degradation every year in the TRHR “wasteland” grassland [63]. Grassland degradation worsens not only soil holding capacity but also soil moisture conditions. Land degradation causes severe water scarcity near the soil surface, and severely degraded grassland has extremely fast water infiltration and poor water retention capacity. The detrimental effect of land degradation on moisture conditions may be greater than expected because the effect will be doubled by a larger active layer thickness due to degradation compared with the experimental warming effect alone [64]. For example, when alpine meadow grassland is degraded, its coverage is reduced, and when weeds replace the original dense-rooted pine grass and grasses, the soil water-holding capacity is substantial reduced, and the soil is dry. Changes in soil and root systems provide higher thermal conductivity, which in turn accelerates soil degradation processes, leading to water infiltration and ultimately a considerable decrease in water conservation [64]. Declining soil water-holding capacity also worsens summer flooding, and future climate change could lead to more frequent extreme flooding in the Yangtze River Basin. Thus, combining the two will worsen things [65].

Grassland degradation is a crucial factor affecting vegetation composition and plant diversity [66]. The species diversity and productivity of TRHR alpine meadows decreased substantially with different degradation stages, and the severe degradation degree led to the migration of alpine meadow plant communities [53]. The degradation of grasslands will undoubtedly lead to the fragmentation of these specific habitats for TRHR. Endangered plant species will be more likely compromised by fragmentation of alpine grasslands, as this will include reducing fragment size and increasing distance to sites with similar habitats [46]. Those species with shorter life cycles may experience greater variation in individual numbers, a combination that could be dangerous for small populations with shrinking habitats, which will undoubtedly lead to a loss of species diversity in grassland ecosystems. Predictably the harsh environment of TRHR makes this sort of thing even more devastating.

**Figure 2:**
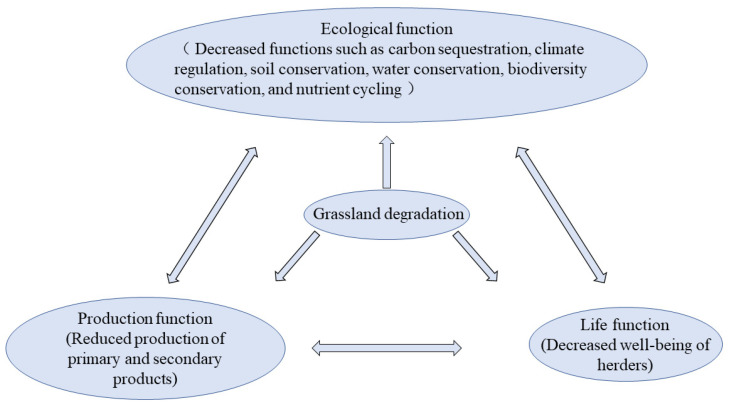
Ecological responses to grassland degradation.

Grassland degradation reduces vegetation cover, height, and productivity, changes in grassland community composition, and allows the invasion of noxious weeds, all of which undermine grassland ecological sustainability [66]. From a production point of view, grassland degradation will undoubtedly impair the yields of primary products (grass, medicinal materials, fungi, and fuel) and secondary products (milk, meat, hair, and hides). From the perspective of the herders’ livelihood, grassland degradation has reduced their well-being, resulting in the emergence of many ecological refugees [58] (Figure 2).

## 4. Driving Factors and Mechanisms of Grassland Degradation

The combination of the fragility of the ecosystem formed by its natural environment and the excessive human disturbance has led to the degradation of grassland on the Qinghai-Tibet Plateau [58]. The bald spots of alpine meadows developed due to the unreasonable use of vegetation, burrowing by rodents, soil loosening, and wind and water erosion [67]. Habitat aridification caused by the bald spots of the grass layer in the alpine meadow prompted the reverse succession of the top plant community dominated by stable Artemisia species. As the area of these bald spots increased, the native vegetation with Artemisia as the dominant species was gradually replaced by poisonous weeds. The alpine meadow was gradually replaced by the bald spot landscape—“black soil beach” [67]. Studies have shown that at the beginning of degeneration, PFG types gradually change from rhizome bunchgrasses to rhizome plexus and dense plexus grasses, then root overgrowth leads to mattic epipedon thickening, the balance between nutrients and water in the soil is broken, the degradation gradually accelerates followed by fissures and collapse of the mattic epipedon [52]. With the gradual intensification of the “hydrothermal hole effect” [67], moisture and heat will be reduced through these hollows, destabilizing the “root–soil–permafrost” system in and around the hollows, creating island-like meadows, and this will accelerate the loss of native forages [68]. With the aggravation of grassland degradation, the spatial heterogeneity of grassland soil nutrients increases, and the soil becomes barren, which is conducive to the invasion of vegetatively propagated poisonous weeds and occupies the “surplus space” left by the high ecological overlap, the poisonous weeds flourish, and have an allelopathic inhibitory effect on other plants [69,70], gradually establishing sustainable populations. In addition, grassland degradation affects the composition and quantity of seeds in seed rain, and its involvement with vegetation; the ability of many weed species to produce large amounts of seed rain leads to further grassland degradation [71]. After establishing sustainable communities, noxious weed species begin to spread nearby or beyond with the help of factors such as climate warming and overgrazing [66]. After severe grassland degradation, the alpine meadow plant community migrates, the response of plant diversity, plant productivity, and soil nutrients mutates, and the grassland loses its ability to restore itself.

The degradation characteristics of different grassland types are dissimilar. Alpine meadow and alpine steppe ecosystems are more stable compared with alpine desert ecosystems and show stronger resistance to disturbance [72]. The underlying mechanism is that the more complex structure and composition of alpine meadow and steppe ecosystems may make them more elastic and more stable. Owing to the influence of the changing environment of natural communities, the changing environmental variables need to be fully considered when evaluating the stability. The relationship between diversity (species and functional diversity) and community temporal stability is perturbation dependent and related to perturbation factors and intensity [73]. Global nitrogen deposition coupled with the increase in CO_2_ concentration may lead to a further decline in the species diversity and functional diversity of alpine grasslands [74,75].

### 4.1. Impact of Climate Change on Grassland Ecosystem

Over the past 60 years, the TRHR has experienced substantial warming, and the rate is higher than that of the global and overall Qinghai–Tibet Plateau [13,37,76,77,78]. Warming varies by season and region. The warming of TRHR since the 21st century is mainly the result of the increase in temperature in the cold season. The annual average maximum and minimum temperature increases in autumn and winter are greater than in spring and summer [79]. From 1982 to 2014, the growing season temperature in the TRHR region showed the most remarkable upward trend in the northwest and southeast [37]. Precipitation has increased slightly in the TRHR region in recent decades [13,76,77]. The regional differences in precipitation increase are evident. Chen, Li, Sivakumar, Li, and Wang [37] showed the areas with substantial increases in growing season precipitation in the TRHR region from 1982 to 2014 were in the northeastern and western parts of the TRHR, with a maximum rate of up to 1.45 mm yr^−1^. Overall, the climate in the region has been in line with the “warm and wet” trend for at least the past few decades [78]. However, studies have also shown the TRHR climate has displayed a “warm and dry” trend in the past 40 years [46]. Other studies have shown that the trend of “warm–dry” or “warm–wet” in the TRHR region varies remarkably in different regions, but it is dominated by warm and wet [80]. In addition, the increasing trend of reference evapotranspiration (ETO) in the TRHR suggests a possible future climate transition to warmer, drier climates [81]. Drought may be the main driver of alpine grassland degradation in the source regions of the Yangtze River and the Yellow River [82]. Climate warming has also caused serious environmental problems. For example, the TRHR region has reported a substantial reduction in the area of permafrost in recent decades, and those areas where the permafrost disappears consistently show the highest temperature increases, resulting in a thinning of permafrost thickness and eventual disappearance [77].

#### 4.1.1. Effects of Climate Change on Plant Growth and Composition

Recent studies suggested climate change may be a major factor in grassland biomass changes [83]. Most recent remote sensing-based model studies have shown that climate change has promoted the growth of vegetation in the TRHR over the past 20 years [84], mainly due to climate warming [13,32,41]. TRHR has a low-temperature, rainy climate, which suggests temperature constrains alpine grassland vegetation more strongly than rainfall. The growth of grassland vegetation benefits from a better thermal environment created by rising surface temperature [32], and permafrost is no longer a limiting factor for root growth. The degradation rate of organic matter is also accelerated. Elevated temperature may increase the photosynthetic rate of grassland, thereby increasing its NPP. Warming temperatures also lead to an earlier start of the growing season of alpine grasslands, which may prolong the vegetation growth period [9] and increase its carbon sink capacity. The regulating effect of temperature on precipitation and cloud cover also promotes vegetation growth [37].

Rising temperatures also have many negative impacts on TRHR grasslands [45]. In the western TRHR, due to poorer moisture conditions, warmer temperatures may increase vegetation ETO, leading to increased moisture constraints on grassland vegetation growth. Studies have shown that climate warming is beneficial to healthy alpine ecosystems, but in degraded meadows in the TRHR, rising temperatures worsen degradation by exacerbating degradation-induced droughts [64], thereby inhibiting meadow plant growth. In addition, the degradation of permafrost, linked to rising temperatures, leads to a drop in the groundwater table, which in turn inhibits the growth of shallow-rooted plants, leading to the degradation of alpine meadows [77,85]. The long-term effect of the deepening of the ecological groundwater level promotes the change of vegetation species and the regional evolution of plants. This situation in turn leads to the degradation and even desertification of alpine grasslands [76]. An increase in temperature may lead to a decoupling between nitrogen and phosphorus [86]. Changes in species dynamics due to warming may also lead to reduced stability [10].

The effect of temperature on grassland productivity is nonlinear, and dissimilarities in different regions of TRHR are evident. The annual average temperature in most grassland areas of TRHR has a positive effect on AGB and NPP [32,45]. Studies have shown the effect of temperature on AG-AGB i is remarkably stronger in the eastern region of TRHR than in the western and southwestern regions. The rise in temperature in the western region of TRHR may worsen the originally severe water conditions due to vegetation ETO, resulting in increased water restrictions on vegetation growth [32]. However, this nonlinear increase and varied responses to temperature increases in different climates suggest the existence of interactions between other non-temperature factors and temperature on plant growth.

Most areas of TRHR belong to a semi-arid and semi-humid climate, and the lack of water severely limits the growth of plants. Precipitation is an important water source for grassland growth and affects the spatiotemporal pattern of AG-AGB. Studies have shown that in the TRHR region, taking 2001 as a node, the temperature and radiation before 2001 were the factors restricting the increase in NPP and then changed to precipitation [84], which highlights the importance of the impact of precipitation on grassland vegetation. Zheng, Han, Huang, Fassnacht, Xie, Lv, and Chen [14] showed that precipitation, rather than air temperature, was considered the key factor responsible for changes in TRHR NDVI values, especially the average precipitation for two consecutive months. In terms of overall vegetation, rainfall is a decisive factor in improving vegetation productivity because increased rainfall can lengthen the growth period of vegetation, which is beneficial to the accumulation of NPP [13]. Furthermore, TRHR precipitation affects the sensitivity of AG-AGB to mean annual precipitation (MAP). If the region has a high drought risk (MAP < 400 mm), the sensitivity of AG-AGB to annual mean precipitation is low, and its change is small. Probably because of the high resistance of vegetation in these arid and semi-arid environments [87], AG-AGB is susceptible to precipitation when MAP varies between 400 and 700 mm, the increase in precipitation is favorable for AG-AGB, In humid areas (MAP > 700 mm), AG-AGB is not substantially affected by MAP, or even negatively affected. This result reflects that sufficient precipitation can promote the growth of grassland leaves and leaf photosynthetic capacity, thereby improving grassland productivity. However, excessive precipitation, whether in saturated or excess conditions, may unbalance soil moisture and gases, affecting plant uptake of nutrients and light, such that increased precipitation does not contribute remarkably to productivity, or even leads to reduced productivity [32]. Zhang, Zhang, Wang, Chen, Gang, An, and Li [84] showed that since 2000, more precipitation in the central region of TRHR has not conducive to plant growth, and high precipitation has led to lower air temperature and radiation [9], which has inhibited plant synthesis of organic carbon. High rainfall also reduces vegetation productivity by increasing topsoil loss, thereby reducing soil organic matter. The responses of NPP and net ecosystem productivity (NEP) of different grassland communities to temperature and precipitation are dissimilar. The NPP of grassland community is remarkably positively correlated with temperature, but the correlation between NPP and NEP and precipitation is low [59]. Nitrogen deposition coupled with increased CO^2^ may also be beneficial to the increase in grassland biomass [74].

Although in the past 20 years, climate change in the TRHR has generally promoted the growth of grassland vegetation [36,37], the dominant factors vary in different regions and periods. For example, the dominant factors in TRHR vegetation growth during 1995–2014 were precipitation in the west, temperature in the southeast and south, and solar radiation in the northeast [37]. Vegetation changes have apparent zonal characteristics, which may be due to the inconsistency in the rate of temperature increase in each region of the TRHR [13]. Although projections showed the vegetation of the TRHR will increase until the end of the 21st century under the RCP_4.5_ climate change scenario [14], the risk of future regional degradation remains due to the potentially abrupt, fragile nature of the TRHR. Future climate change may affect community stability by causing desynchronization among alpine grassland populations [10]. Furthermore, the increasing trend of ETO in the TRHR suggests a future climate transition to a warmer, drier climate will accelerate the transition from grassland to heathland, especially in the high-altitude regions of the mid-west of TRHR [81]. With the improvement of grassland degradation level in this area, the proportion of weeds increases, with the trend of warm and humid climate, the invasion rate of weeds further accelerates [66].

#### 4.1.2. Impact of Climate Change on Grassland Plant Phenology

The main drivers of plant phenological changes are air temperature and precipitation. Grazing and nitrogen deposition may also affect phenological changes [88], and snowfall may also play a key role in the actual growing season [89,90], but the phenological responses vary with snow cover duration, grassland types, and temperature and precipitation gradients [91,92]. Studies have reported that since the 1980s, many alpine grasslands have generally experienced an early start and delayed end of the growth period, which has prolonged the growth period [88,93]. Piao et al. [94] suggested the increase in the length of the growing season in alpine meadows from 1982 to 1999 may be due to the increase in temperature. To reveal the related mechanism of phenological change further, some scholars also studied the thermal growth season change of TRHR, and then compared it with the actual growth season. The thermal growing season is closely related to temperature changes. Liu, Zhu, Pan, Zhu, Zhang, and Zhang [89] used TRHR in 1960–2013 daily temperature data and field observation phenology data to show the temperature increase in winter and spring is a key factor affecting the early start of the actual growing season. For the actual growing season, the early end of the season is affected by the increase in summer temperature, whereas the delay of its end is affected by the increase in summer precipitation. Some studies have also found that the advance of the TRHR vegetation growing season is most strongly affected by the average temperature from March to May, with an advance of 1.97 days for every 1 °C increase, whereas the end of the growing season is related to the cumulative sunshine length from August to September, and a 10-h extension can lead to an advance of 0.07 days [93]. However, Liu, Zhu, Pan, Zhu, Zhang, and Zhang [89] have shown that although the duration of the thermal growing season has become longer due to rising temperatures, the duration of the actual growing season may not have increased, but rather advanced overall. The earlier start of both is associated with the response to temperature increase, whereas the later end of the thermal growing season is associated with the earlier end of the actual growing season. One of the possible explanations is that due to the short growth period of grassland in this area, the increase in thermal energy can accelerate the completion of the growth cycle. Another possible explanation is that warm summers lead to water scarcity and inhibit grassland growth, but the actual situation should be complex and variable [95,96]. Another study on the source of the Yellow River showed soil thaw onset is the major factor leading to the advance in the SOS of alpine meadow regions [97]. In addition, the effects of temperature and water on phenology have noticeable interactions, and the sensitivity of grassland plant phenology to temperature increase is also regulated by water conditions [88]. Owing to the regional differences in TRHR climate and its changes, regional differences may exist in its phenological responses. Changes in the phenology of TRHR grassland ecosystems may, in turn, affect the interactions between grassland plant species, resulting in changes in ecosystem structure and impacts on ecosystem functions such as carbon and water cycles.

#### 4.1.3. Impact of Climate Change on Soil

Climate warming increases ETO and aggravates surface water infiltration, which gradually induces land degradation in TRHR alpine meadows, eventually leading to topsoil drought [64]. The increase in air temperature makes the soil temperature rise and the permafrost thaw, and the eco-hydrological process of the source area changes. Soil moisture infiltrates the root zone due to the thawing of the permafrost, which eventually leads to poor soil moisture status [77,98]. Recent studies have found nitrogen deposition coupled with increased CO_2_ may also lead to soil drying [74]. Wang et al. [99] showed an increase in the likelihood of soil damage due to increased rainfall and runoff erosion over the past few decades, coupled with warming. Therefore, the situation may worsen in the future.

Climate change may be a major factor affecting SOC changes [83]. Climate change negatively affects soil carbon and nitrogen pools [100]. Changes in soil organic carbon density (SOCD) of TRHR are sensitive to temperature increase. SOCD in TRHR showed remarkable interannual variation from 1981 to 2010, but the trends over 30 years were not consistent [101]. The downward trend in SOCD after 2003 was associated with climate warming and increased spatial heterogeneity of precipitation. After 2003, the decrease in precipitation in the east often led to the decomposition of SOC, whereas the increase in precipitation in the west led to a relatively stable SOC. Further predictions showed that due to the warm–wet trend of the future climate in the TRHR, heterotrophic respiration will increase and may overtake plant production during 2011–2070, leading to a decrease in SOC. After 2071, the increment of NPP will be higher than that of heterotrophic respiration, and SOCD will start to rise, especially in the west. The simulated SOC responses to climate change also have substantial regional differences. The decrease in SOCD in the east and the increase in the west may be caused by the regional differences in carbon input produced by plants. The carbon input in the western region may increase, but the carbon input in the eastern region remains relatively unchanged or even decreases [101]. Recent studies have also shown that nitrogen deposition will accelerate the decomposition of SOC in alpine grasslands, affecting soil carbon pools [102].

### 4.2. Effects of Grazing on Grassland Ecosystems

With the rapid development of the TRHR agricultural economy since the 1980s, overgrazing, grassland abandonment, and construction have all had a remarkable impact on ecological processes, resulting in grassland degradation, habitat loss, and landscape fragmentation [84,103]. These effects substantially affect the ecological welfare of downstream residents [7]. Moreover, grazing partially offsets the positive contribution of climate change to grasslands [37]. Many researchers agree overgrazing is the main cause of grassland desertification [11,104].

#### 4.2.1. Status of Overgrazing

Recent studies showed that although the grazing pressure in TRHR decreased in the past 20 years, it is still in a state of overgrazing [105,106,107], and further reducing the number of livestock in TRHR is necessary to alleviate the grazing pressure [1,104,108,109,110]. Around 2015, all winter pastures in the TRHR region were overgrazed. However, only 37.5% of the summer pasture area was overgrazed [106]. Grazing pressure on winter pastures is much higher than on summer pastures because these pastures are closer to settlements and watering facilities. Longer grazing time and greater grazing intensity usually result in more degraded pastures in winter than in summer [108,111]. Furthermore, the degree of overgrazing varies in different counties. Zhang, Zhang, Liu, Qi, and Wo [105] showed that in 2010, the total number of overgrazing sheep in TRHR was 652 × 10^4^ sheep units (SU), the average overgrazing rate was 67.88%, and the average overgrazing number was 27.43 SU·km^−2^. Tongde, Xinghai, Yushu, Henan, and Zeku had higher overgrazing rates; Zhiduo, Golmud, Dari, Qumalai, and Maduo had no overgrazing; while the rest of the counties had overgrazing. Zhang, Zhang, Liu, and Qiao [1] showed the grazing population in the TRHR area exceeded the carrying capacity by 132,800 people from 2000 to 2015, especially in counties such as Xinghai, Tongde, Zeku, Yushu, Nangqian, and Chengduo. In addition, the balance between grassland carrying capacity and livestock and wildlife is critical to maintaining the stability of grassland ecosystems [104]. The ratio of domestic animals to wild ungulates in the TRHR area has been estimated to be approximately 4.5:1 [109,110]. A study in Maduo County showed that grasslands are mildly overloaded when only livestock are considered but moderately overloaded when livestock and large wild herbivores are considered. Therefore, if large wild herbivores are not considered when calculating the forage balance, grazing pressure will be underestimated by about 22% [110]. In addition, the division of national park functional areas has a substantial impact on the balance of grass and livestock in the Yellow River Source National Park. After the implementation of the zoning plan, the grassland in the Yellow River Source National Park remained overloaded. If the number of grazing livestock remained unchanged, the grazing pressure doubled, and the conflict between pasture and livestock became more evident [112].

#### 4.2.2. Effects of Grazing on Plants

Grazing reduces AG-AGB (Lu et al., 2017). Recent studies have shown that moderately grazing yaks remarkably reduces the maximum height of adult grassland vegetation [113]. Fencing substantially increases above-ground vegetation productivity [114]. Under long-term enclosure conditions, the total AGB of fenced and non-grazing grasslands is higher than that of free-grazing grasslands. This result suggests fencing management can promote plant biomass, especially herb biomass and belowground biomass in the uppermost 0–10 cm [115]. Vegetation shifts to low, sparse types due to excessive consumption and trampling of grassland by livestock [60]. Different species also respond differently to dissimilar grazing intensities [116].

Grazing also affects plant composition and diversity. Vertebrate herbivores control diversity by altering species composition, including the invasion of non-native species and the extinction of native species [117]. Herbivores can maintain plant diversity, as grazing benefits native flowering plants and increases ground light [118]. The disturbance intensity determines the composition and diversity of grassland species in different pasture types. Fencing reduces plant species diversity [115], but long-term fencing is beneficial to the improvement of forage functional groups and inhibits the development of harmful weed functional groups [114]. Mild and moderate grazing intensities can promote plant diversity [72] and nectar production in alpine grasslands due to increased numbers of florets and flower heads, reduced competition for light, and increased numbers of flowering individuals per plot [119], which supports moderate perturbation hypothesis. The plant diversity–biomass–cover relationship on alpine plateaus may be decoupled by overgrazing livestock [120], and overgrazing creates bare patches that provide suitable habitats for receiving weed seed rain and cultivating weed seedlings. However, some studies found the stability of the ecosystem does not change under different grazing intensities because the recovery and resistance of plant functional groups vary in grazing responses [116]. On the impact of grazing practices on grass species diversity, studies found that multifamily grazing pastures (pastures are not fenced and are grazing by multiple households) have higher species richness than single-family grazing pastures (pastures are separated by fences and are grazing by single households) because single-family operations may concentrate sustained, higher grazing pressure on small areas of grassland, leading to a reduction in plant diversity [121].

Grazing not only affects species richness, but also adversely influences soil moisture and TN. It also indirectly increases the density of spring seed bank and decreases the density of summer seed bank through these effects. Recent studies showed that grazing regimes change the species composition of the vegetation, but the seed bank composition does not change much. Although seed bank size does not change much with grazing intensity, it reduces the number of persistent seeds [122], which are important for grassland restoration.

#### 4.2.3. Effects of Grazing on Soil

Grazing alters soil properties by altering plant functional group composition, biomass loss, and nutrient cycling in alpine grassland ecosystems, with considerably negative effects on soil physical and nutrient properties. Grazing intensity is one of the key factors affecting soil properties in grassland ecosystems [123]. In recent decades, TRHR soil erosion acceleration was remarkably associated with livestock numbers and intensive grazing, but not with precipitation. Grazing is more important than climate change for soil erosion. Overgrazing reduces vegetation cover and fine roots, and accelerates soil erosion [124]. Grazing activities have a negative impact on soil moisture retention. Grazing reduces the moisture content in the upper 30 cm soil layer of alpine steppe, alpine meadows, and temperate steppe, especially in the 10–20 cm soil layer of alpine meadows [125]. The upper soil hardness and pH of different grazing treatments in the alpine meadow ecosystem showed an increasing trend with increasing grazing activity, but a substantial difference in hardness was observed between the summer pasture and winter pasture grazing treatments. Grazing had a substantial effect on soil total phosphorus and available phosphorus content. With the increase in grazing intensity and the increase in total potassium and available potassium, SOM, SOC, and TN decreased substantially [117], and the C/N ratio showed a similar law. Soil properties such as soil carbon and nitrogen generally decrease with increasing grazing intensity, possibly due to the increased turnover of plant matter and litter [126], as well as physical damage to soil, and accelerated soil C and N loss due to high grazing intensity [123]. Similarly, Fan, Hou, Shi, and Shi [115] showed the total carbon and C/N ratios in the aboveground tissues of fenced and ungrazed grasslands are considerably higher than those of free-grazing grasslands.

### 4.3. Influence of Plateau Pika on Grassland Ecosystem

**Figure 3:**
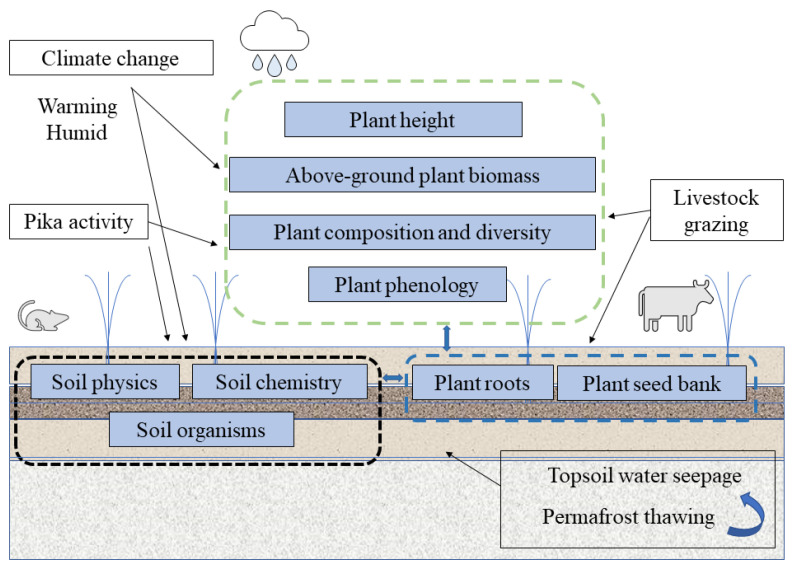
Effects of climate change, livestock grazing, and pika activity on grassland ecosystems.

Pika activity is also one of the factors contributing to TRHR grassland degradation. Studies have shown that pika activity reduced soil moisture, hardness, SOC, and TN [127]. The AGB, species number, cover, and leaf area index of the grassland decreased with increasing pika density. Higher cave densities reduce net ecosystem CO^2^ exchange, gross ecosystem productivity, and ecosystem respiration [128]. Some studies showed disturbance in pika is more important than grazing [129]. However, Yi et al. [130] showed pika consumes 8–21% of the average annual NPP of alpine grasslands, and the effect of pika mound on the reduction of vegetation cover, biomass, soil carbon, and nitrogen is much smaller than that of bald spot, all less than 10%. Large voids in pristine grasslands are the result of strong root systems, whereas those in new and old pika mounds and bald patch soils are smaller and discontinuous. Water generally preferentially passes through soil macropores, whereas in new and old pika mounds and bald patches more readily occurs at the surface, resulting in topsoil loss [131]. In addition, fine-grained soils loosened by pika activity can be blown away by frequent strong winds, thereby increasing the proportion of gravel in the soil [128], increasing soil erosion, and hindering vegetation restoration [127]. Coupling of overgrazing and rodent infestation may lead to the formation of bare patches, ultimately severe degradation of TRHR grasslands [132] (Figure 3).

Many differences still need to be unified in the understanding of the driving mechanism and response consequences of the TRHR alpine grassland ecosystem. Furthermore, there are many uncertainties in future climate change, which also increases the complexity. Future research should aim at a more comprehensive, clearer understanding of the driving mechanisms and response consequences, as this should be an effective means for us to deal with future risks. Sufficient systems knowledge to underpin future grassland conservation, which requires more on-site ecosystem-level studies, is still lacking. Remote sensing is an important method for monitoring vegetation and land, and related studies are many [133]. However, due to the complexity of ecosystem processes and the limitations of remote sensing data, its full potential has not been realized, and data fusion methods should be used to overcome these limitations further [134]. Therefore, the combination of field experiments and modeling will be an important direction for future research.

## 5. Restoration of Degraded Grasslands

### 5.1. Protection Policy of Grassland Ecosystem

Since 2000, due to the obvious adverse effects caused by grassland degradation, the government and researchers have gradually realized the importance of TRHR grassland protection, and formulated a series of environmental protection policies, which have achieved remarkable results [2,135,136,137]. To achieve effective management, TRHR environmental protection projects should pay more attention to the integration of market-based logic in the formulation of conservation policies. However, the market mechanism emphasized in the actual policy design is not reflected in practice because of the absence of a well-integrated scientific measurement standard, lack of accountability mechanism, and weak supervision and implementation. Furthermore, this negatively affects pastoralists due to the coercive nature of registration. The effectiveness of market logic-based environmental policies can be harnessed by using more scientific metrics, more liberal registration, and subsidizing money based on actual restoration results of grasslands [138]. Incentive policies have an enhanced interaction effect on economic benefits and environmental value on herders’ satisfaction with grassland ecological protection and incentive policies. Given that herders pay more attention to substantive subsidies and rewards [139], policy formulation should focus more on the adjustment of market prices, the implementation of incentive policies, and the promotion of emerging technologies to guide the rational use of land, promote rational use of land, and ensure the safe, sustainable development of ecosystems [140]. In addition, helping residents of TRHR deeply identify with grassland restoration plans and use conservation techniques is important [141] because their participation is largely dependent on the benefits they receive and their perception of the benefits of the program. Therefore, the successful implementation of the policy can be ensured through education and the use of incentive policies [141]. Furthermore, the trigger and feedback of family decision-making are very important and need enough attention, which has important practical importance for the protection of alpine grassland [142]. Managers should also enhance their understanding of the relationship between ecological and cultural conservation in the TRHR region, which will improve future ecosystem management and cultural conservation because successful management cannot happen without acknowledging the local Tibetan people and their traditional customs and culture as part of conservation. Resource management of any kind is essentially about how to manage sustainably while preserving, and even improving, the lifestyles and cultures of those who harvest and use the resources. Any concern about well-being and poverty are grounded in local realities, considering the most relevant aspects (environment and people affected) across multiple dimensions and, different groups’ responses to policies [143].

### 5.2. Restoration Measures for Degraded Grassland

#### 5.2.1. Adaptation to Climate Change

Owing regional differences in geographical background, climate change, hydrothermal conditions, and conflicts between pastures and livestock, differences in the types, degrees, scales, and time courses of regional grassland degradation are great [144]. The ecosystem services provided by different regions and their variations are also quite different [145]. Therefore, planning ecosystem restoration projects in a targeted manner according to the regional differences in the functions and needs of each region is very important [144,145]. The trend of increased ETO observed in summer months poses a threat to the growth of natural vegetation, suggesting TRHR requires more irrigation, which has been determined to have a greater impact on vegetation than the observed decrease in ETO in winter. Therefore, more measures will be needed in the future, such as artificial rainfall, to counteract the negative effects during the summer months [81]. In addition, studies show that recovering new species assemblages that have emerged due to climate change is very difficult, if not impossible [146]. According to the trend analysis of grazing ability, climatic factors have a remarkable impact on the grazing ability of TRHR, and the grazing potential has changed drastically. Therefore, selecting and adjusting strategies in a timely manner, optimizing livestock breeds, and selecting grass species that are more adaptable to climate change are necessary. In addition, various preparations must be made for climate change as early as possible, and artificial intervention mechanisms developed to enable the healthy development of animal husbandry.

Ecological restoration measures should be prioritized in areas with relatively warm, humid climates, where grassland productivity will be greater [147]. However, from the perspective of the fragility of the ecosystem, arid regions also need to prioritize the implementation of ecological restoration measures [108,148]. Promoting the use of solar energy in households is also important for improving ecosystem functioning and adapting to climate change. The use of solar energy equipment provides more manure to the grassland, thereby greatly improving the ecosystem service value of the grassland, especially the alpine grassland in this area [149]. In general, further comparative studies and mechanism analysis are essential to formulate better ecological restoration strategies in TRHR to cope with the uncertainties of future environmental changes [108].

#### 5.2.2. Sustainable Grazing Management

Moderate grazing is a good way to maintain grassland biodiversity and, the function of grazing ecosystem, and develop the productivity of grassland ecosystem. Rational use is the best protection strategy [150], and this requires precise management driven by information and technology. Ref. [151] recently showed that sensors and Internet technology can be used to obtain forage-related information and livestock behavior. On the one hand this decision support system provides great potential for future precision grazing management. On the other hand, from the overall perspective of the region, based on the carrying capacity of the grassland, the yield of grass, the number of livestock, and the number of herders should be kept in balance [150,152]. Continuing to reduce livestock, increasing the slaughter rate, controlling the number of livestock, easing the grazing pressure, promoting scientific breeding methods, and changing the traditional rough grazing mode are necessary. In addition, regulating the herder population, carrying out population transfer, or directly realizing the transformation from traditional animal husbandry to other industries are needed [1]. Improved management efforts should go directly to cool-season pastures, and adjusting the proportion of seasonal grazing area is also an effective alternative strategy that would optimize carbon and nitrogen sequestration [153], which will achieve a “win–win” for grasslands and households [106]. Owing to the deterioration of the balance between supply and demand in animal husbandry, planting forage crops or using highland barley varieties for crop rotation is an effective way to alleviate the imbalance between supply and demand of forage grass from the perspective of supply [154]. In addition, the input–output balance of feeding systems is very important for livestock health and output, and further attention needs to be paid to the importance of nutrients and antinutrients [155]. Studies based on grassland sensitivity and impacts showed that efforts to improve grassland adaptive capacity should be based on increasing the area of fenced pastures, warm sheds, and sown grasslands, and reducing livestock densities as well as strengthening TRHR ecological engineering protection [156].

#### 5.2.3. Restoration of “Black Soil Beach”

Diagnosis of grassland conditions is an important first step in grassland ecosystem management. The effectiveness of meadow restoration through long-term efforts is strongly related to the level of degradation [157]. Depending on the degree of degradation, different measures should be taken [158]. Some studies have found that biotic drivers of degraded alpine grassland vegetation may be more important than abiotic drivers [159]. Therefore, to prevent grassland degradation, the primary task is to carry out reasonable grazing management on non-degraded grassland. For mildly and moderately degraded grasslands, implementing rodent control, limiting grazing to low stocking levels, and using fencing, weeding, fertilization, and scratching the turf techniques for grassland restoration are recommended [152]. However, long-term fencing needs to be cautious, as it may lead to adverse effects on plant diversity and soil enzyme activity [160].

However, for the severely degraded “black soil beach”, grazing prohibition and rodent control alone cannot restore severely degraded grasslands, and even if the stressors are removed, restoring the grassland ecosystem to its original state is impossible. Recent studies have shown that “black soil beach” is dominated by perennial weeds, which are more difficult to remove than annual weeds. The restoration of “black soil beach” by fencing and abandoning tillage increases the stability of secondary plants in degraded grasslands of “black soil Beach”, which is not conducive to the restoration of “black soil Beach” [152]. Therefore, feasible protection and restoration measures need to be taken to prevent the alpine grassland from further degrading into a collapsed “black soil beach ” state, that is, an irreversible state of grassland degradation. Targeted human interventions, including selective planting of pasture and artificial sowing of grassland to cope with the “inertness” of alpine grassland as well as ecological and biological control of high-altitude rodent populations are recommended to restore “irreversibly” degraded pastures [146]. Studies have also shown that weed species richness decreased as native forage grasses were artificially seeded, altering the plant composition of “black soil beach” in the short term [161]. “Black soil beach” showed visible changes after three years of restoration, and the quality of forages increased with the increase in restoration time [162]. Xu et al. [163] showed that restoration behaviors (restoration of planting and enclosure) improved AG-AGB weakly, but substantially promoted species richness, target species richness, and target species AGB. This situation means grassland cultivation can accelerate the restoration of degraded grasslands, allowing target species and communities to be established and developed.

The restoration of “black soil beach” should also be intervened from the perspective of the vegetation–soil system. Restoring the soil carbon and nitrogen storage in the alpine grassland through vegetation reconstruction on the “black soil beach” takes more than nine years, and the C and N storage changes in a “V” shape with the vegetation restoration time [100]. Scientific management of soil nitrogen availability during restoration and succession can delay the occurrence of secondary degradation of vegetation and grassland [164]. During grassland reconstruction, proper planning is needed to enhance soil carbon and nitrogen storage potential, which is essential to maintain the healthy development of the ecosystem. Soil management is an integral part of grassland restoration, and this is indirectly supported by the fact that belowground biodiversity plays an important role in community stability, directly and indirectly [165]. Wu, Zhang, Gao, Xu, Wu, Shan, Liu, Dong, Dong, and Wen [161] showed that the restoration of artificial grasslands can achieve good results after 8 years; after 16 years, moderate control measures should be taken to guide the restoration and succession; while after 18 years, the species composition of artificial grassland is close to the natural state. Plants, soils, and plant–soil systems exhibit nonlinear resilience of artificially reconstructed grasslands along a temporal gradient. Plant resilience is highest in the 12th year. After 13 years of revegetation, revegetated grassland soils outperforms severely degraded grasslands. The elasticity of soil and plants are not synchronized in the time gradient. Plant–soil system resilience is highest in the 16th recovery year. Therefore, from a system–wide perspective, the rebuilding time for severely degraded level grasslands will take at least 16 to 18 years to stabilize. Although restored grasslands can be relatively stable after 16 to 18 years of reconstruction, the restored ecosystems are still much lower than healthy alpine meadows in terms of plant and soil quality [166]. After 4 years of artificial planting and restoration of degraded grassland, the number of unrelated species in the community decreases, the community’s sensitivity to external disturbances increases, and a trend of reverse succession is observed. The development of arable grassland leads to an increase in neutral interactions among plant species with prolonged recovery time. The proportion of positively and negatively correlated species decreases. At the later stage of recovery, the niche occupied by a single species is narrow, and species coexist harmoniously, reaching a relatively stable state [162]. Therefore, in the long run, continuous monitoring should be carried out, monitoring accuracy and accuracy should be further improved, and appropriate manual intervention should be taken according to the situation to prevent the secondary degradation of artificial grassland [61]. In addition, using near-natural restoration methods to restore alpine grasslands with different degrees of degradation may be a more effective, sustainable restoration method, providing a nature-based solution for grassland restoration [158].

## 6. Conclusions

The monitoring of grassland ecological information is the basis for effective management. This review shows that in terms of monitoring TRHR grassland dynamics under changing environments, NDVI-based grassland dynamic analysis urgently needs to use more complex methods to adapt to the complexity of the ecosystem and the uncertainty of vegetation dynamics under climate change to explore the nonlinear relationship of vegetation dynamics fully. Second, current research on grassland cover modeling focuses on the exploration of machine learning models for higher accuracy, and many studies have identified the advantages of SVMA methods in cover data simulation. Finally, for AG-AGB monitoring, most studies show that RF models generally exhibit higher stability and accuracy than other machine learning models, and more research on AGB process models is needed. This review also shows that the monitoring of grassland ecological information needs to strengthen the consideration of the grassland species structure, so hyperspectral data can be used to achieve a clearer understanding of grassland ecological changes.

TRHR grasslands have been degraded to varying degrees in the past 50 years. Although grassland coverage and AG-AGB showed an overall increasing trend after 1990; this recovery did not reflect the overall or fundamental improvement, grassland degradation has not been fundamentally curbed, and the risk of future regional degradation still exists. Grassland degradation is a very complex ecological process, including changes in vegetation and soil. The assessment of grassland emphasizes the importance of analyzing the soil–plant system from the perspective of a multidisciplinary strategy and constructing a reasonable monitoring system for grassland degradation simulation. Once grasslands are graded for degradation, adjusting their use according to our degradation system will help prevent irreversible degradation of important grasslands. The ecological effects of TRHR alpine grassland degradation can be reflected in ecology, production, and livelihoods. Grassland degradation greatly weakens ecosystem functions such as carbon sequestration, climate regulation, soil conservation, water conservation, biodiversity protection, and nutrient cycling, and affects grassland production functions and herders’ livelihoods. Grassland degradation remarkably reduces surface soil nutrients and greatly affects the distribution of SOC, the resulting loss of SOC, and a positive feedback on climate warming. The degree of grassland degradation is proportional to the intensity of soil erosion. Grassland degradation worsens soil moisture conditions, and the effects of this deterioration may be greater than previously thought. Grassland degradation leads to not only the decline of grassland species diversity and productivity, but also the fragmentation of specific habitats, which will undoubtedly accelerate the loss of species diversity. The adverse effects of degradation on the yield of primary and secondary products will also reduce the well-being of pastoralists.

The TRHR climate has generally shown a “warm and wet” trend in recent decades, and climate change has generally promoted the recovery and growth of alpine grasslands. Overgrazing is common in all counties and townships of TRHR and is considered by many researchers as one of the main reasons for grassland degradation. However, climate change and livestock grazing activities have a very complex impact on the grassland ecosystem of TRHR, related research is still controversial, and further research is needed to unify these differences. Grassland changes and the dominant factors of the changes in different regions of the TRHR in different periods are dissimilar, and the uncertainty of changes will further increase under the background of climate warming. Attention must be paid to the research on the driving mechanism of grassland change, and quantitative studies on the impact of climate change and human activities on the grassland ecology of TRHR, which are crucial for the protection of grassland ecosystems, are still relatively few. Moreover, a clear understanding will increase the likelihood of successful restoration of degraded grasslands. Therefore, the research on grassland change and its influencing factors is undoubtedly a process of continuous efforts. In addition, the impact of pika on the TRHR grassland needs to be evaluated further to determine the degree of impact on the grassland ecosystem to determine the status of the pika.

The TRHR ecological protection policy needs to emphasize the effective integration of market logic, consider the feedback and triggers of family decision making, and help herders understand the restoration plan and master restoration techniques through education and publicity to achieve all-around effective management. Conservation policies also need to enhance awareness of the relationship between ecological and cultural conservation in TRHR and acknowledge that local Tibetans and their traditional customs and cultures are part of the conservation, so that successful management can be achieved.

The grazing capacity of TRHR has changed drastically due to climatic factors. In response to this, grazing capacity should be effectively regulated, better livestock breeds should be selected, and grass species more adaptable to climate change should be cultivated. In addition, various preparations for climate change must be made as early as possible, and artificial intervention mechanisms must be formulated to enable the healthy development of local animal husbandry. Rational utilization is the best strategy for grassland protection. The likelihood of success of long-term grassland restoration strategies depends on the level of grassland degradation, and different measures should be taken according to the degree of degradation. The effectiveness of rehabilitating meadows through cyclical efforts depends on the degree of degradation, and different measures should be taken depending on the degree of degradation. Implementing rodent control, light grazing, enclosure, weeding, fertilization, and other techniques for restoration of grasslands with mild and moderate degradation levels is recommended. However, severely degraded “black soil beach” needs to be restored by artificial seeding. Appropriate manual intervention is required to prevent secondary degradation during the restoration succession. Research in grassland restoration needs to emphasize the stability of plant–soil systems to establish relatively stable communities.

## Figures and Tables

**Table 1:** Abstract of the research on dynamic monitoring of TRHR grassland.

	Study Area	Dataset Source (Periods)	Main Method	Results	Reference
Grassland Monitoring and Simulation Based on NDVI	TRHR	1. GIMMS 3g. v1 (1982–2015) 2. Institute of Remote Sensing and Digital Earth, Chinese Academy of Sciences (2000–2015) 3. Landsat NDVI date (1982–2015)	-	1982–2015, NDVI increased slightly; 2000–2015, >70% NDVI increase	Bai, Guo, Degen, Ahmad, Wang, Zhang, Li, Ma, Huang, Zeng, Qi, Long and Shang [13]
	TRHR	MODIS VI (MOD13Q1) (2001–2016)	-	Increased NDVI in most grasslands	Ge, Meng, Liang, Feng, Gao, Yang, Huang and Xie [18]
	TRHR	Pathfinder database (Pathfinder Data Sets) of U.S. Earth Resources Observation Systems Data Center (1982–2006)	-	1994–2004, NDVI increased; 2004–2006, especially substantial	Qian, Fu and Pan [15]
	TRHR	Ten-day MODND1T NDVI product	DBEST	Vegetation generally recovered, although the progressive fluctuations of trend observed	Shen, An, Feng, Ye, Zhu and Li [17]
	TRHR	GIMMS 3g. v1 (1982–2015)	-	No remarkable change in NDVI, and it increased in the west and decreased in the east	Gillespie, Madson, Cusack and Xue [16]
	TRHR	GIMMS AVHRR NDVI datasets (2000–2013)	-	From 2000 to 2013, NDVI decreased slightly. Projected to increase by 2100 under RCP_4.5_ climate change scenario	Zheng, Han, Huang, Fassnacht, Xie, Lv and Chen [14]
	TRHR	MODIS (MOD09GA) (2003–2014)	-	NDVI gradually increases from northwest to southeast	Liang, Yang, Feng, Liu, Zhang, Huang and Xie [19]
Monitoring and Modeling of Grassland Cover and Grassland Aboveground Biomass	TRHR	MODIS (2001–2016), the measured grassland cover data collected by unmanned aerial vehicle (2014–2016)	SVM	The average annual maximum grassland coverage increases from west to east and from north to south; 59.9% annual maximum grassland coverage increases	Ge, Meng, Liang, Feng, Gao, Yang, Huang and Xie [18]
	Qumalai, Gande, Henan, Xinghai, Tongde	Fields observation (1994–2006)	-	Grass height increased from 1994 to 2006; dry biomass of Qumalai, Gande, Xinghai increased, and Henan decreased, and all four sites increased substantially from 2004 to 2006; Gande, Qumalai grassland coverage increased, Xinghai changed slightiy, and Henan decreased	Qian, Fu and Pan [15]
	Zhiduo, Qumalai	HJ-1A/HIS and Landsat 8 (16 September 2013), Fields observation (August 2017)	RF, SVM	Native plant coverage was generally higher than noxious weeds	Ai, An, Chen and Huang [23]
	TRHR	Fields Observation (2003–2014), MODIS (MOD09GA) (2001–2016)	BP-ANN	The largest AGB appeared in the easternmost and north-central regions; 2001–2006, TRHR had more AGB increasing areas than decreasing (44.4% and 29.2%, respectively), and 26.4% stable areas. AGB in the far east (Zeku, Henan) and far southwest (part of Golmud and Yushu) increased substantially (16.5%), while AGB in the northwest of Zhiduo County decreased significantly (3.8%)	Yang, Feng, Liang, Liu, Zhang and Xie [25]
	TRHR	MODIS (MOD09GA) (2003–2014), Field Observation (2003–2014)	Multifactor regression analysis	AG-AGB gradually increases from northwest to southeast	Liang, Yang, Feng, Liu, Zhang, Huang and Xie [19]
	Headwater of the Yellow River	MODIS (MOD13Q1) (2001–2020), Field Observation (2005, 5006, 2015, 2018, and 2020)	RF	Headwater of the Yellow River AGB decreased from southeast to northwest; 2001-2020, 69.51% area increased, 30.14% area decreased	Tang, Zhao and Lin [31]
	TRHR	Field Observation (2005–2016), MOD13Q1 (V006, NDVI and EVI included) (2000–2018)	RF, Cubist, ANN, SVM, and Bayesian model averaging (BMA)	AGB was higher in the southeast and lower in the northwest; 2000–2018, the interannual variation trend of grassland in most areas was not evident	Zeng, Ren, He, Zhang, Li and Niu [32]

Note: TRHR: Three-Rivers Headwaters Region, NDVI: normalized difference vegetation index, AG-AGB: aboveground biomass of alpine grassland, HYR: Headwater of the Yellow River, DBEST: Detecting Breakpoints and Estimating Segments in Trend.

## Data Availability

Not applicable.
